# Horizontal mitochondrial transfer as a novel bioenergetic tool for mesenchymal stromal/stem cells: molecular mechanisms and therapeutic potential in a variety of diseases

**DOI:** 10.1186/s12967-024-05047-4

**Published:** 2024-05-24

**Authors:** Roberto Iorio, Sabrina Petricca, Vincenzo Mattei, Simona Delle Monache

**Affiliations:** 1 Department of Biotechnological and Applied Clinical Sciences, University of L’Aquila, Via Vetoio, 67100 L’Aquila, Italy; 2 Dipartimento di Scienze della Vita, Della Salute e delle Professioni Sanitarie, Link Campus University, Via del Casale di San Pio V 44, 00165 Rome, Italy

**Keywords:** Mitochondria, Horizontal mitochondrial transfer, Mesenchymal Stromal/Stem cells, Tunnelling nanotubes, Extracellular vesicles, Neuronal diseases, Ischemic vascular diseases

## Abstract

Intercellular mitochondrial transfer (MT) is a newly discovered form of cell-to-cell signalling involving the active incorporation of healthy mitochondria into stressed/injured recipient cells, contributing to the restoration of bioenergetic profile and cell viability, reduction of inflammatory processes and normalisation of calcium dynamics. Recent evidence has shown that MT can occur through multiple cellular structures and mechanisms: tunneling nanotubes (TNTs), via gap junctions (GJs), mediated by extracellular vesicles (EVs) and other mechanisms (cell fusion, mitochondrial extrusion and migrasome-mediated mitocytosis) and in different contexts, such as under physiological (tissue homeostasis and stemness maintenance) and pathological conditions (hypoxia, inflammation and cancer). As Mesenchimal Stromal/ Stem Cells (MSC)-mediated MT has emerged as a critical regulatory and restorative mechanism for cell and tissue regeneration and damage repair in recent years, its potential in stem cell therapy has received increasing attention. In particular, the potential therapeutic role of MSCs has been reported in several articles, suggesting that MSCs can enhance tissue repair after injury via MT and membrane vesicle release. For these reasons, in this review, we will discuss the different mechanisms of MSCs-mediated MT and therapeutic effects on different diseases such as neuronal, ischaemic, vascular and pulmonary diseases. Therefore, understanding the molecular and cellular mechanisms of MT and demonstrating its efficacy could be an important milestone that lays the foundation for future clinical trials.

## Background

Mitochondria are highly dynamic and multifunctional compartments that play a pivotal role in oxidative bioenergetic metabolism and in maintaining cellular homeostasis. In addition to energy production, mitochondria perform many other key functions regulating fatty acid β-oxidation, iron homeostasis, Ca^2+^ metabolism, heme and steroid hormones biosynthesis, innate immunity, redox homeostasis, and cellular waste management [1–3]. They are key sensors of multiple types of cellular stress [4–6] and crucial determinants in cell survival and death [7]. To fulfill its multiple tasks, mitochondrial network is dynamically redesigned by distinct processes (collectively referred to as mitochondrial quality control, MQC), including fission and fusion events, intracellular mitochondrial movement, selective removal of damaged mitochondria through mitophagy [8–10], and mitochondrial biogenesis, that orchestrate the overall shape, size, distribution, and connectivity of mitochondria. As signaling platforms, mitochondria communicate extensively with other cellular compartments [11] and can operate outside their intracellular confines exchanging information between cells, and even across organ systems [12]. Interestingly, different forms of circulating mitochondria (e.g., wrapped Mitos, mitochondria transported in vesicles or in platelets; free Mitos, mitochondria without a protective vesicle membrane) with distinct effects on immune cells have been found in both blood and cerebrospinal fluid (CSF) [8, 13–16].

It is not surprising that the concomitant increase of mitochondrial DNA (mtDNA) mutations and reactive oxygen species (ROS) generation, during the aging process, exacerbate mitochondrial dysfunction and dysregulates MQC, contributing to the pathogenesis of multiple age-associated diseases [17, 18], recently termed as non‐communicable diseases (NCDs) [19].

In this view, mitochondria-targeted therapeutic approaches have been explored over the past decade as potential treatments for tissue revitalization and homeostasis.

The phenomenon of intercellular transport of mitochondria between mammalian cells, also known as horizontal transfer of mitochondria, has recently attracted a renewed attention from the scientific community, representing an intriguing reparative strategy [20, 21]. Intercellular mitochondrial transfer (MT) is a novel form of cell-to-cell signalling involving the active incorporation of healthy mitochondria into stressed/injured recipient cells [22], contributing to restore the bioenergetic profile (ATP and mitochondrial membrane potential) and cell viability, to increase the mtDNA content, as well as to reduce inflammatory processes and normalize calcium dynamics [23].

**Fig. 1:**
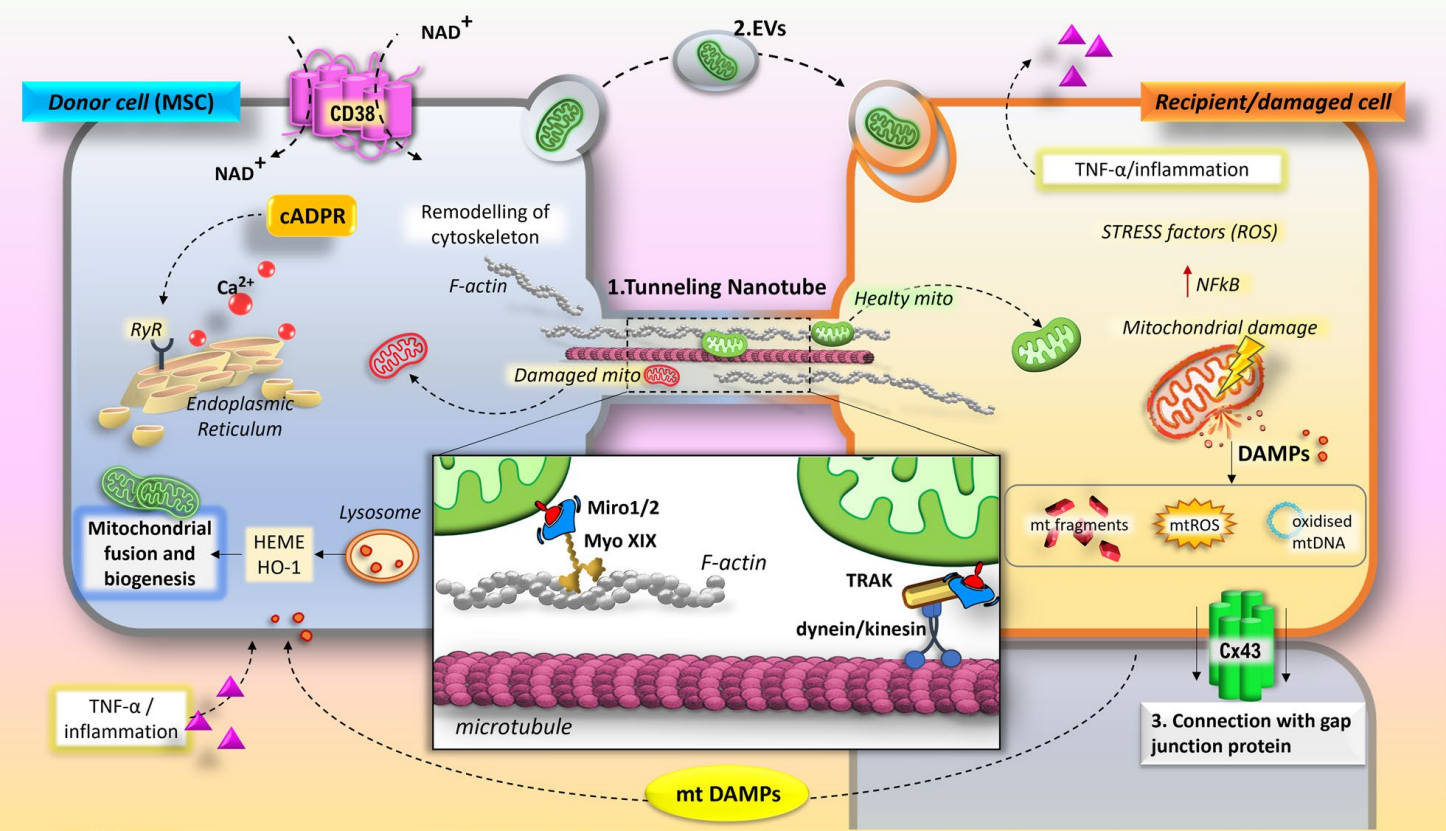
Different mechanisms and routes of intercellular mitochondrial transfer. Exposure to stress signals (e.g., dysfunctional mitochondria, mitoDAMPs, and NAD^+^) triggers the transfer of healthy mitochondria from donor cells to stressed/injured recipient cells via the activation of distinct signaling pathways and different routes, such as TNTs (1), EVs (2), and GJs (3). Key participants in the development of TNTs are F-Actin, microtubule, and intermediate filaments. Microtubule-based mitochondrial movement is mediated by Miro/TRAK complex attached to KIF5 kinesin/dynein molecular motors, whereas myosin motors are involved in mitochondrial transport based on actin. The NAD^+^/CD38/cADPR/Ca^2+^ pathway is involved in the release of EVs. In the presence of NAD^+^, CD38 can synthetize cADPR through its cyclase activity. As second messenger, cADPR triggers the release of the intracellular Ca^2+^ from the Endoplasmic reticulum by acting on Ryanodine Receptor (RyR). Therefore, the calcium-mediated activation of exocyst complex leads to the release of vesicles into the extracellular space. Importantly, the endocytosis of EVs may also occur via these mechanisms. Cx43-GJs are often located to one end of specific TNTs and the transcellular transfer of mitochondria may occur via gap junction internalization

MT can occur via multiple distinct molecular mechanisms, including tunnelling nanotubes (TNTs), extracellular vesicles (EVs), gap junction channels (GJCs), and other non-traditional routes, such as cell fusion and mitochondrial extrusion [24, 25] (Fig. 1).

The mitochondrial donation from mesenchymal stromal/stem cells (MSCs) has gained increasing attention in recent years [26], suggesting its potential in stem cell therapy [21]. MSCs-mediated MT is emerging as a critical regulatory mechanism for cell and tissue regeneration, and damage repair, where a remarkable restoration of cellular bioenergetics and a reduction in oxidative stress have been reported [26, 27]. MSCs, as reported by Viswanathan et al., are to be better referred as Mesenchymal Stromal/Stem Cells (MSCs) given the prevalence of stromal cells with respect to the smaller fraction of true "mesenchymal stem cells” (10.1016/j.jcyt.2019.08.002). MSCs have the ability to maintain tissue homeostasis and renewal via regulating functional activity of parenchymal cells, including haematopoietic stem cells (HSCs) [28, 29]. They can be isolated and expanded in vitro from almost all tissues, including bone marrow (BM), dental pulp (DP), umbilical cord (UC), adipose tissue (AD), placenta, and other sources [30–33]. Moreover, they have pleiotropic ability to differentiate into mesodermal lineage cells, including adipocytes, chondrocytes and osteocytes, or ectodermal lineage cells, such as neuronal and neuroglial cells [34]. In physiological conditions, the original microenvironment characteristics of MSCs (intercellular matrix, other cell types, soluble factors and humoral components) dictate their cellular fate [35]. On the other hand, MSCs reciprocally interact with the microenvironment through distinct processes, including immunomodulation and tissue repair. Therefore, MSCs display remarkable tissue regenerative properties given their ability to receive distinct signals from the surrounding tissues (e.g., damage-associated molecular patterns (DAMPs) and ROS) and constantly set up intricate intercellular communication networks with stressed/damaged cells.

Mitochondrial exchange and its beneficial effects were firstly reported by Spees and colleagues [36] in A549 ρ° cells with mtDNA deletion or defects. Specifically, the acquisition of healthy mitochondria in A549 ρ° cells following active interaction with human MSCs resulted in increasing levels of oxygen consumption, membrane potential, and intracellular ATP. Subsequent investigations in different in vitro and in vivo models have revealed the possibility of using mitochondria derived from MSCs for therapeutic purposes (e.g., stroke, lung and acute respiratory disorders, brain injury, muscle sepsis, and cardiac diseases), thus representing a novel strategy to treat many NCDs. Features such as immune privilege, significant migratory capacity to injured sites, fine-tuned redox balance, and low oxidative damage levels, as well as low energy demand (mitochondria in MSCs are quiescent and exhibit low activity level) render MSCs as elective donor cells in delivering functional mitochondria to diseased cells [37–42]. Furthermore, MSCs isolated from different tissue types (BM; AD; DP; UC; Wharton’s jelly, WJ) exhibit distinct bioenergetic signatures that influence their MT capacity, with AD-MSCs and BM-MSCs showing higher MT levels to cardiomyocytes than DP-MSCs and WJ-MSCs [37].

On the other hand, it has also been shown that MSCs-mediated MT plays a critical role in pathological states. In this sense, recent evidence indicates a dark side of MSCs in increasing malignancy of cancer cells, supporting tumor microenvironment and cancer progression, and providing metabolic flexibility and chemotherapy resistance [37, 43–45].

In this review, we highlight the therapeutic potential of MT from MSCs in restoring the bioenergetic metabolism and cell functionality/viability into stressed/injured recipient cells. Therefore, we provide a comprehensive overview of the molecular signals triggering MT, as well as the major routes and mechanisms mediating MT. Finally, recent advances in MSCs regenerative properties through MT process in different NCDs are proposed.

## Intercellular mitochondrial transfer via multiple cellular structures and mechanisms

MT has been demonstrated in various contexts under physiological (e.g., in tissue homeostasis and stemness maintenance) and pathological conditions (e.g., hypoxia, inflammation, and cancer). MT is a highly regulated multistep process that requires the spatiotemporal orchestration of many factors, including distinct triggering events/signals, molecular processes governing the formation of transfer machinery, and regulatory elements that modulate mitochondrial transfer speed and duration. In this section we will discuss the current state of knowledge regarding molecular stress signals and modes of MT, as well as signaling pathways regulating the different patterns of mitochondrial exchange.

### Mitochondrial transfer trigger signals

Depending on different forms and activation states, mitochondria can affect the functions of neighbouring cells performing complex activities as mediators of regenerative and anti-inflammatory effects or trigger factors of inflammatory reactions [8, 46–48]. Multiple stress conditions, including oxygen–glucose deprivation [49, 50] drug-induced oxidative stress [51, 52] and inflammation [53, 54] generate harmed and fragmented extracellular mitochondria that trigger MT between cells. Therefore, the tissue regenerative properties of MSCs via the acquisition of active mitochondria by injured cells may be prompted by stress signals such as dysfunctional mitochondria and DAMPs of mitochondrial origin (mitoDAMPs), including ROS, DNA, cardiolipin, ATP, N-formyl peptides, transcription factor A mitochondria (TFAM), Cytochrome C (Cyt-C), succinate, and Ca^2+^(Fig. 1).

Consistent with this hypothesis, in an in vivo model of myocardial infarction, mitochondria released from damaged cells operate as potential DAMPs in MSCs, inducing the activation of eme-oxygenase-1 (HO-1) signaling pathway and mitochondrial biogenesis. These events enhance MT and consequently potentiate the rescue ability of MSCs to damaged cells [55]. In acute myeloid leukemia (AML) cells, NADPH oxidase-2 (NOX2)-derived ROS regulates MT from the BM-MSCs to the AML through TNTs [56]. In line with this, the generation of oxidative stress in HSCs triggers MT from BM-MSCs. Specifically, infection by Gram-negative bacteria drives MT from the BM-MSCs to HSCs through a ROS-dependent mechanism involving the activation of p53 and its downstream Akt/PI3K/mTOR pathway, and Connexin 43 (CX43) Gap Junctions [57, 58]. Of note, the p53-dependent activation of Akt/PI3K/mTOR pathway also leads to the overexpression of Tumor Necrosis Factor Alpha Induced Protein 2 (TNFαip2) and the formation of TNTs. As described above, MSCs also promotes the transfer of depolarized mitochondria to macrophages in response to ROS generation [58].

In addition to ROS, the Cyt-C released in stressed cells can also stimulate MT. Therefore, in UV-damaged PC12 cells at early stage of apoptosis loss of Cyt-C from injured mitochondria activates MT to healthy cells through TNTs formation, thereby leading to the recovery of apoptotic PC12 cells [59].

CD38 is a multifunctional transmembrane glycoprotein responsible for the biosynthesis of two calcium-mobilizing second messengers, cyclic ADP-ribose (cADPR) and nicotinic acid adenine dinucleotide phosphate. Interestingly, CD38 may also play a role in promoting MT in different cellular models. Therefore, by a calcium-dependent mechanism involving CD38 and cADPR signalling, astrocytes promote the mitochondrial exchange to nearby neurons via microvesicles (MVs), thereby contributing to activation of neuroprotective and neurorecovery mechanisms after ischemic stroke [60]. In this case, CD38 expression may be related to the excessive release of excitotoxic glutamate from ischemic neuron, suggesting for this neurotransmitter a potential role in promoting astrocytes-mediated MT. A subsequent study further demonstrated that CD38/cADPR signaling and mitochondrial Rho GTPases (Miro) 1 and 2 contribute to the MT between astrocytes, and from neuronal cells into astrocytes [61]. CD38 has been indicated to exert a critical function during bone formation [62]. Very recently, it has been demonstrated that CD38/cADPR signaling plays a crucial role in mediating the differentiation and maturation of osteoblasts and osteoprogenitor cells through stimulating the secretion of mitochondria and mitochondrial-derived EVs [63].

### Tunnelling nanotubes (TNTs) as a major route for mitochondria exchange

Multiple in vitro and in vivo models have highlighted the elective role played by TNTs in MT. Specifically, MT can occur from MSCs to lung macrophages via TNTs, resulting in higher phagocytic activity of macrophage cells in a model of acute respiratory distress syndrome [64]. In an in vitro simulated ischemia/reperfusion model, MSCs move mitochondria to injured H9c2 cardiomyoblasts via TNTs, protecting cardiac cells against the apoptosis [65]. A bidirectional MT between MSCs and vascular smooth muscle cells via TNTs upregulates MSCs proliferation [66]. Also, MT via TNTs occurs from human BM-MSCs and endothelial cells experiencing chemotherapy stress, resulting in recovery of injured endothelial cells [67]. Mitochondria move between BM-MSCs and myeloma cells via TNTs, as described above [56, 68]. TNTs-mediated mitochondria exchange also form between donor cells other than MSCs, including PC12 cells [59], astrocytes [69], lung epithelial cells [70], cancer cells [71], retinal pigment epithelium [72], and trabecular meshwork cells [73].

#### Structural features of TNTs

According to the initial description, TNTs are open-ended membranous channels directly connecting cytoplasm of cells over long distances in a homotypic and heterotypic fashion. They are non-surface adherent and have a structure of 50–900 nm (with an average of 200 nm) in width and an average length between 20 and 100 mm.

TNTs contain F-actin cytoskeletal filaments that allow the bidirectional and unidirectional exchange between cells of various‐sized cargoes, including small molecules, nucleic acids and proteins (e.g., tau, α-synuclein, and huntingtin), organelles (e.g., vesicles, lysosomes, endoplasmic reticulum (ER), mitochondria, and autophagosomes), and even virus (herpesvirus and SARS-CoV-2) and bacteria [74–78], suggesting their role in in coordinating metabolism and signalling events in a wide-range of physiological processes and pathological conditions. Of note, this transfer function represents a crucial distinctive characteristic between TNTs and filopodia. The membrane structure of TNTs is very heterogeneous and cell type-specific. Therefore, close-ended TNTs (exhibiting gap junctions), that allow transfer of electrical signals, have also been identified in different in vitro and in vivo conditions [72, 79–81]. In this regard, 3 types of close-ended TNTs have been observed, such as “*hand-shake*”, “*invaginated*”, and “*resting*” [82].

In addition, two types of open-ended TNTs according to the different distribution of cytoskeletal elements have been described: “thin” TNTs (< 700 nm in diameter), containing only F-Actin, and “thick” TNTs (> 700 nm in diameter), with F-Actin, microtubule, and intermediate filaments [83]. In neuronal and stromal cell lines, cryo-correlative light and electron microscopy (cryo-CLEM) coupled with tomography have also revealed individual TNTs (iTNTs), a bundle of small open-ended tubes (up of two to 11) that run parallel, and sometimes braided together, along the entire length of the TNTs [82, 84]. These structures exhibit N-Cadherin as molecular linker connecting adjacent iTNTs. In addition to confer mechanical stability, it acts as guidance during growing iTNTs. Differently from single TNT, iTNTs allow for a bidirectional transfer of cargoes, including vesicles and mitochondria.

#### Mechanisms of TNT formation and protein regulators

Thus far, we have learned that the formation of TNTs can be generated via two different mechanisms that can occur simultaneously, or change dependently on the environmental conditions, though the underlying molecular process still remains under active investigation. These mechanisms include the “*cell dislodgement”,* wherein cells initially in contact leave behind a tubular connection when they move apart, and the “*protrusion-elongation mechanism*” where the cell extends a filopodia-like protrusion to another cell located at some distance. In this regard, the main mechanism responsible for TNTs biogenesis seems to be cell dislodgement, as reported by live imaging analyses in different cell types [85, 86]. However, the protrusion-elongation mechanism is typical of post-mitotic cells with low migratory phenotype, such as neurons and epithelial cells [87].

As mentioned above, the biogenesis of TNTs involves stress-signaling (e.g., p53 and MAP kinase) and pro-survival (e.g., EGFR, Akt, ROCK, PAK, MAP/ERK, PI3K or mTOR) pathways. Downstream effectors of these signaling pathways are proteins related to both membrane recycling and cytoskeletal remodeling.

Interestingly, the Rab family of small GTPases regulates many steps of membrane trafficking and also participate in actin cytoskeleton remodeling [88, 89]. Therefore, transport and recycling of vesicles regulated by the small GTPases Rab11a and Rab8a promote TNTs formation in different cell systems [88, 90]. Specifically, VAMP3 (vesicle-associated membrane protein 3, often called v-SNARES) operates downstream of Rab8a to regulate TNTs biogenesis [88]. Depletion of Ras association domain-containing protein 1A, a master regulator of cellular homeostasis and cytoskeleton, results in Rab11 accumulation and the subsequent release of exosome, thereby leading to TNTs formation [91]. Furthermore, Rab35 and its downstream effectors, such as ACAP2, ARF6-GDP, and EHD1 operate in a cascade mechanism to promote TNTs biogenesis in neuronal cells [92].

F-actin remodeling processes are responsible for initiating TNT protrusion by acting against the tensile strength of the plasma membrane, thus allowing it to deform and generate forces for tube growth. In this regard, distinct mechanisms involving specific sets of actin regulatory proteins are implicated, such as M-Sec (also known as TNFAIP2) [93–95] the exocyst complex, leukocyte specific transcript 1 (LST1) [96], the unconventional Myosin X (Myo10) [97], the actin bundler epidermal growth receptor substrate 8 (Eps8) and insulin receptor tyrosine kinase substrate protein 53 kDa (IRSp53), and small GTPases (e.g., Miro1/2, Rac1, Cdc42 and RalA) [98].

The cytosolic protein M-Sec acts as a key regulator of TNTs biogenesis through interaction with the small GTPase Ral in the mouse macrophage-like cell line RAW264.7 [93]. In this context, the transmembrane MHC class III protein LST1 may operate as a membrane scaffold for the generation of multi-molecular complex that controls the formation of TNTs. Thus, LST1 promotes TNTs formation by recruiting RalA and the actin-crosslinking protein filamin to the plasma membrane. It also stimulates the binding of RalA with two components of exocyst complex, Sec5 and Exo84, thereby leading to actin cytoskeletal remodeling and membrane protrusion. Meanwhile, the interaction of LST1 with M-Sec, myosin, and myoferlin may be involved in the process of mitochondrial anchoring and transfer [96]. In this regard, the recruitment of cytosolic M-Sec to the plasma membrane during the initial phase of TNT formation may occur through its direct binding to phosphatidylinositol (4,5)-bisphosphate [PI(4,5)P_2_] or phosphatidylinositol (3,4,5)-trisphosphate [PI(3,4,5)P_3_] [94]. Nucleolin, an RNA-binding protein, is essential for TNT formation in various types of mammalian cells [99]. Specifically, the binding of nucleolin to 14-3-3ζ mRNA modulates phospho-cofilin levels, leading to its inactivation, F-actin polymerization, and TNTs biogenesis.

In addition to the Rab35 signaling, *Wingles-related integration site* (Wnt)/Ca^2+^ pathway, an intracellular cascade that is implicated in actin cytoskeleton remodeling, promotes TNTs biogenesis and stability in CAD (mouse catecholaminergic neuronal cell line) cells and primary neurons through the modulation of the interaction between the β isoform of Ca^2+^/calmodulin‐dependent protein kinase II and actin [100].

The I-Bin/Amphiphysin/Rvs (BAR)-domain protein IRSp53 is an essential spatio-temporal coordinator of plasma membrane protrusions (through promoting negative membrane curvature) that couples Rho-GTPase signaling to cytoskeleton remodeling and membrane dynamics. As a signaling platform, IRSp53 operates under the control of activatory (Cdc42, Eps8) or inhibitory (14-3-3) inputs as well as downstream effectors, and recruits to the plasma membrane various actin regulatory proteins, such as Eps8, Ena/VASP4, Wiskott-Aldrich syndrome protein (WASP), N-WASP, mDia2, WASP family verprolin-homologous 2 (WAVE2), and others [101, 102].

**Fig. 2:**
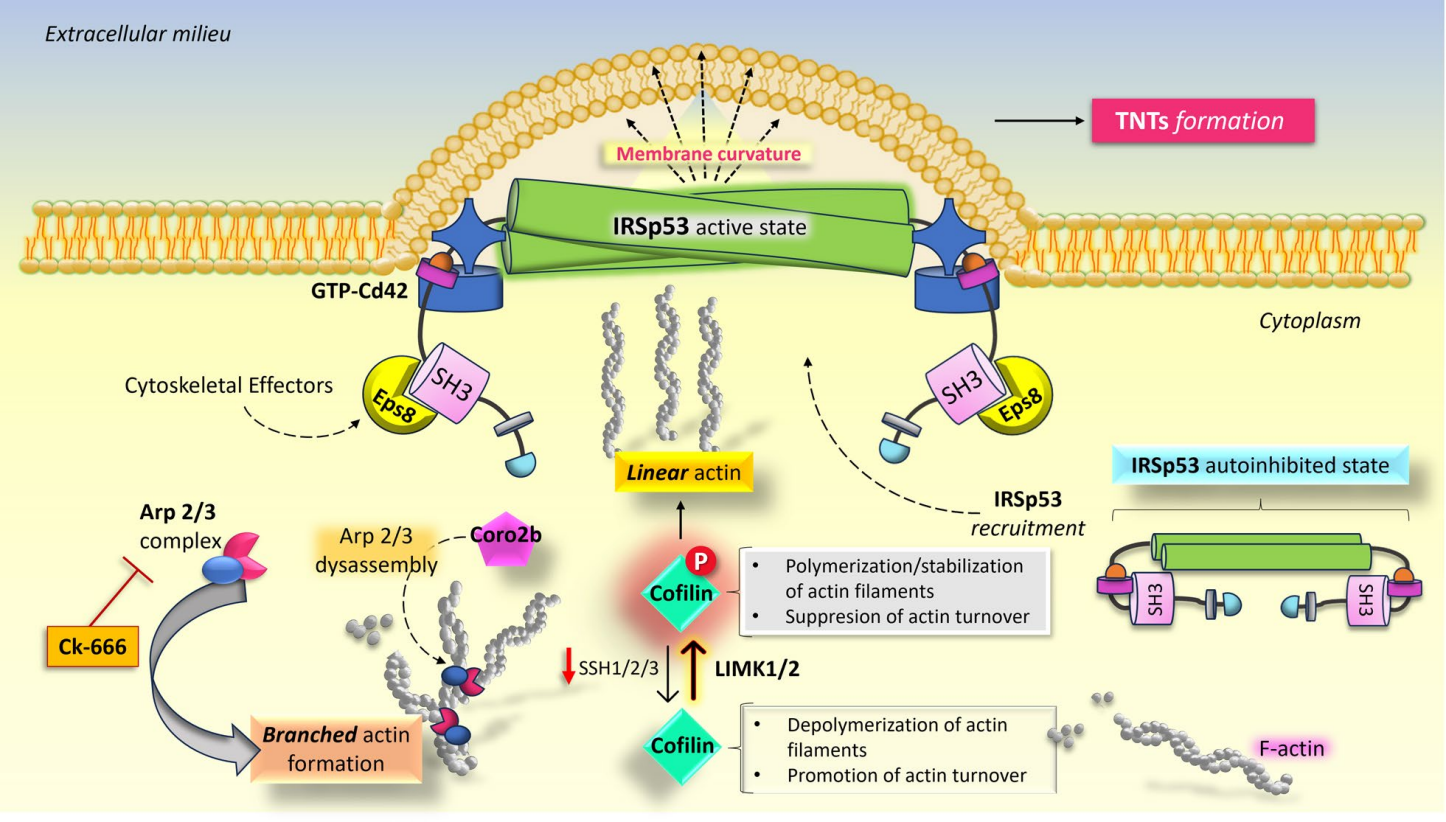
Current model of TNTs formation via Eps8-IRSp53 interaction. In the cellular environment, the assembly of actin can be organized into two general types of architectures in balance with each other, such as branched actin networks and closely packed parallel arrays. The inhibition of Arp2/3-dependent branched filaments enhances the synergic activity of Eps8-IRSp53 complex at the membrane causing the shift of the actin balance in favour of polymerization of long, linear filaments for TNTs outgrowth. In this context, Eps8 seems to play a crucial role, possibly through renewed interactions with proteins and signaling pathways involved in actin dynamics, including phospho-regulators of cofilin (e.g., slingshot protein phosphatases and LIM-kinases), and coronins (e.g., Coro2b). Therefore, changes in Eps8’s interactome would lead to drastic reduction of actin filament turnover and disassembly of Arp2/3 networks, while promoting the extension and organization of actin

Therefore, Delage and colleagues [98] have shown that Cdc42/IRSp53/VASP network negatively regulates TNTs formation and vesicular transport in neuronal cells, suggesting that Cdc42-dependent pathways are mainly involved in filopodia formation, rather than TNTs generation. By contrast, Eps8 (that reduces filopodia formation) is a positive regulator of TNTs biogenesis as its overexpression leads to increases in the extent of TNT connections and cargo transfer. Interestingly, the inhibition of actin related protein 2/3 complex (Arp2/3; a seven-subunit protein complex that mediates the formation of branched networks of filamentous actin, crucial for both filopodia and lamellipodia formation) promotes TNTs biogenesis and actin polymerization over longer distance in neuronal cells [82]. In particular, Arp2/3 inhibition enhances the co-expression of Eps8 and IRSp53, thus favoring their synergistic interaction. This event promotes the reorganization of the actin cytoskeleton, thereby leading to the switch of the system towards the extension of straight F-actin formation rather than the formation of branched networks [87] (Fig. 2). In addition, another recent study has demonstrated that the inhibition of ROCK, a downstream effector of Rho/Rac/Cdc42, stimulates TNTs formation via Myosin II mediated F-actin modulation [103].

However, the same Rho GTPase signaling pathways operating in neuronal cells may act in a different way depending on the cell type. Indeed, observations in macrophages indicate that the blockage of Arp2/3 results in a decrease of TNTs formation [104]. Moreover, pathways converging on Arp2/3, including Cdc-42-WASP and Rac1-WAVE2 contribute together to TNT biogenesis. Consistent with this, in human trabecular meshwork cells (HTMCs; endothelial-like cells), the inactivation of Arp2/3 complex causes a reduction in TNT number and length, as well as vesicles transfer [73]. On the contrary, disassembly of actin stress fibers formation using Rho kinases inhibitors increased the number of TNTs and vesicle transfer.

Myo10 is an unconventional myosin that has critical functions in TNTs and filopodia formation [97, 105]. In particular, neuronal TNTs biogenesis requires both the N-terminal head (the myosin motor domain that can bind to F-actin) and C-terminal tail domains (that can bind to several molecules, such as PI(3,4,5)P_3_, microtubules and β-integrins) of Myo10. Myo10 operates downstream of Cdc42 and promotes TNTs formation independent of VASP (a strong inducer of dorsal filopodia) and through a mechanism independent of integrins and substrate attachment, suggesting that Myo10-driven TNTs may arise from a different mechanism than the one that drives the formation of dorsal filopodia [98, 105, 106].

In neurons, the extracellular protein S100A4 and its receptor RAGE (Receptor for Advanced Glycation End Product) play crucial role in directing TNTs biogenesis [107]. Under oxidative stress, p53-caspase-3 axis determines the cleavage and depletion of S100A4, thereby leading to a concentration gradient between neurons and astrocytes that drives the extension direction of TNTs from neurons (with low concentration of S100A4) to astrocytes (with high concentration of S100A4).

#### Role of Miro proteins in mitochondrial transfer via TNTs

Miro 1 and 2 proteins are embedded in the outer mitochondrial membrane and involved in the structure of the mitochondrial contact-site and cristae organizing system complex (that connects outer and inner-mitochondrial membrane), Ca^2+^ metabolism, mitochondria-ER communication, and mitophagy. Besides these functions, Miro proteins have critical role in regulating the mitochondrial spatial positioning and transport acting as adaptors that link mitochondria to cytoskeleton-associated motor proteins [108, 109]. Generally, microtubules and actin microfilament system mediate long- and short-distance transport of mitochondria, respectively. In this regard, movement of mitochondria based on microtubules involves the interaction of Miro/TRAK (Trafficking Kinesin Protein) complex with different motor proteins, such as Kinesin, dynein, and kinesin superfamily KIF5 [110, 111], while the actin-based mitochondrial movement is mediated by myosin family members, including myosin II, V, VI, and XIX [112, 113].

Recent studies demonstrate the essential role of Miro proteins in TNTs‐mediated mitochondrial [38] transport [114–118]. Specifically, overexpression of Miro1 promotes MT from donor MSCs to epithelial injured cells, leading to the rescue of epithelial functions. On the contrary, Miro1 knockdown inhibits MSC-mediated MT [114]. Consistent with this, upregulation of Miro1 in MSCs increases the metabolic/bioenergetic benefits of MT following neuronal oxidative stress and mitochondrial damage, while decreasing Miro1 expression reduces these effects [115, 117]. Also, MT via TNTs from astrocytes to neurons rescues neurons from cisplatin-induced damage. Furthermore, siRNA-mediated knockdown of Miro1 in astrocytes reduces MT, thus preventing the normalization of neuronal calcium dynamics [116].

### Mitochondrial transfer via gap junctions (GJs)

MT can also occur in a Cx43-dependent manner since the deletion of Cx43 in donor/acceptor cells usually negatively affects the process [119–121]. Canonically, the formation of GJs or hemichannels across the plasma membrane by six Cx43 monomers allows uni- and bidirectional transfer of ions, small molecules (e.g., glucose, prostaglandins, microRNAs, and secondary messengers), and organelles between cells, thereby regulating intracellular mechanisms of signaling and several cellular functions [50, 122]. In line with this context, the MT from BM-MSCs to injured alveolar epithelium requires the Ca^2+^ exchanges between the two cells via Cx43-GJs, in the lipopolysaccharide (LPS)-induced acute lung injury mouse model [119]. In addition, MT from hematopoietic progenitors to BM-MSCs is required to induce the metabolic recovery of recipient BM-MSCs following irradiation and is cell-contact dependent and mediated by Cx43 [121].

Besides this classical function, Cx43-GJs may play further physiological roles, such as the regulation of mitochondrial functions and mediation of MT through the formation of TNTs and GJs internalization. In particular, Cx43 has also been detected within mitochondria [123] and seems to play a crucial role in mitochondrial calcium homeostasis and cell survival [124]. Cx43 is also implicated in forming connections between TNTs [125, 126] and marked reduction of TNTs is observed following knocking out the Cx43 genes in human trabecular meshwork [127]. Also, Cx43-mediated TNTs formation is crucial for MT from human induced pluripotent stem cell (iPSC)-derived MSCs to the injured bronchial epithelial cells and results in the inhibition of asthma inflammation [120]. Importantly, a novel mechanism for MT mediated by Cx43-GJs has been reported [128]. Specifically, this process requires a distinct form of GJs turnover involving the engulfment of GJs (a form of trogocytosis). Thus, following GJs internalization whole mitochondria and endosomes are incorporated into vesicles (named connexosomes/annular gap junctions) and transferred between neighbouring granulosa cells.

### Mitochondrial transfer mediated by extracellular vesicles (EVs)

EVs are heterogeneous, phospholipid-enclosed structures that play a pivotal role in cell-to-cell communication at a longer range [129]. They are released by any cells into the extracellular space under different physiological and pathological conditions and encompass small EVs or exosomes (diameter of 30–200 nm), microvesicles (MVs or ectosomes; diameter of 100–1000 nm), and apoptotic bodies or apoptosomes (> 1 μm in size) [130, 131] Very recently, a specific subset of small EVs of mitochondrial origin has been identified in mouse and human brains named “mitovesicles” [132–134]. Mitochondria encapsulated into EVs can be transferred between cells to maintain the survival of metabolically compromised cells [58], to regulate immune responses [135], or to maintain tissue homeostasis [136]. In a mouse model of focal cerebral ischemia, MT by EVs between astrocytes and neurons acts as a survival mechanism protecting neurons from glucose deprivation and hypoxia [60]. EVs-mediated MT from renal scattered tubular cells (STC-like cells) to injured tubular epithelial cells (TEC) exerts protective effects, resulting in attenuating renal stenosis and recovering mitochondrial respiration [137]. Very recently, it has been demonstrated that mitochondria containing MVs (diameter of about 185 nm) derived from a human brain microvascular endothelial cell line significantly increase ATP levels, mitochondrial respiration, and glycolytic capacities of the ischemic primary human brain microvascular endothelial cells [138]. Interestingly, EVs-based interorgan transport of mitochondria may protect the heart through hormetic responses [139]. Specifically, circulating EVs derived from energetically stressed adipocytes, and containing oxidatively damaged mitochondria are taken up by cardiomyocytes, where they promote oxidative stress, resulting in a metabolic and redox adaptation of the heart that may confer protection against a future lethal stress.

In in vitro models of acute respiratory distress syndrome (ARDS), EVs-mediated MT from MSCs to human macrophages promotes phagocytosis and abolishes proinflammatory cytokine secretion [e.g., TNF-α] by macrophages, thereby mitigating lung injury [140]. Furthermore, in a model of allergic airway disease, myeloid-derived regulatory cells (MDRCs) encapsulate and transfer mitochondria to peripheral T cells via EVs, thereby affecting their bioenergetic and/or redox profile [141, 142]. Active mitochondria (in EVs and isolated) released by platelets stimulates the pro-angiogenic activity of MSCs via metabolic remodeling, including increased de novo fatty acid synthesis [143]. In this context, the clathrin-dependent endocytosis is the mechanism by which mitochondria are internalized by acceptor cells. Accordingly, EVs from MSCs are emerging as a novel nano-strategy approach to attenuate mitochondrial damage and improving TFAM-mtDNA complex stability, essential for regenerative capability of different tissues [144].

### Other routes of mitochondrial transfer: cell fusion, mitochondrial extrusion, and migrasome-mediated mitocytosis

Although most data to date suggests that MT can occur via TNTs, Cx43-GJs, and EVs, various other routes have been proposed, including cell fusion, mitochondrial extrusion, and the migrasome-mediated mitocytosis [21, 145].

Cell fusion involves the physical merging of two or more cellular membranes, which would hypothetically allow the exchange of multiple protein complexes and even organelles, such as mitochondria [146]. Although cell fusion is infrequent under normal conditions, it may result in a marked mitochondrial delivery into recipient cells following injury and inflammation [147], hypoxia-induced apoptosis [148], or irradiation [149]. Cell fusion between human MSCs and cardiomyocytes results in MT into cardiomyocytes, and the resulting hybrid cells are reprogrammed toward a progenitor-like state [150]. Stem cells can fuse with other cellular models, including hepatocytes [151] and neurons [152], generating hybrid phenotypes which summarize distinct characteristics of both cells [153].

MT can also involve the extrusion or internalization of free mitochondria or mitochondrial components without membranous carriers. Under stress conditions, these events are crucial for regulating mitochondrial turnover and homeostasis [145, 154, 155]. Uncouplers of oxidative phosphorylation strongly stimulate the complete release of fragmented mitochondria from highly glycolytic HeLa cells via a mitoptosis process [154, 156, 157]. An analogous mechanism is also confirmed in in vivo models, including cardiomyocytes [158] and neurons [159, 160] suggesting a possible role as “waste removal” [145]. In *Caenorhabditis elegans*, under proteotoxic stress, neurons release dysfunctional mitochondria and protein aggregates via large membrane‐bound vesicles called “exophers” to support cellular homeostasis and functionality [160]. Murine cardiomyocytes can remove mitochondria and a significant portion of subcellular components via exopher‐like structures that are finally taken up and eliminated by cardiac macrophages. This process would support heart homeostasis by preventing extracellular accumulation of waste material, autophagic block, and inflammasome activation [158].

In hepatocytes and fibroblasts, the extrusion of mitochondria is also promoted by LPS through a process resembling secretory autophagy [161]. TNF-α induces the release of naked mitochondria into the extracellular spaces [155]. Similarly, massive mitochondrial extrusion has been shown in cell models of TNF‐α-induced necroptosis [162]. In this regard, free mitochondria would act as a specific danger signal to trigger inflammatory processes. Therefore, activated platelets can also release active mitochondria, both within membranous carriers and as free compartments, able to induce inflammation [163].

Importantly, MT may also occur via migrasomes, vesicular structures that grow on the tips and intersections of retraction fibers of migrating cells via tetraspanin microdomains [164, 165]. Therefore, to maintain cellular homeostasis, damaged mitochondria would be transported to the cell periphery before disposal by mitocytosis, an important mitochondrial quality-control process [166]. This process would deliver damaged mitochondria to surrounding cells [167].

## Mesenchymal stem cells-mediated therapeutic effects of MT on different diseases

MT-mediated by MSCs may be used in restoring the bioenergetic metabolism and cell functionality and may be a useful tool for several diseases’ treatment. The MSCs potential therapeutic role has reported in several papers with a proposed mechanism which suggests that MSCs may enhance tissue repair after injury by MT and shedding of membrane vesicles [168]. MT is a strategy investigated in several kinds of cells such as pulmonary, cardiac, renal, corneal epithelium, and brain cortical [37]. Rustom et al. reported mitochondrial donation via a new form of cell-to-cell interaction based on TNTs [169]. Moreover, Jang et al. described intercellular mitochondrial transportation from MSCs to corneal endothelial cells, photoreceptors, and retinal pigment cells [170] and that recipient cells exhibited increased mitochondrial respiratory abilities. In the same paper Jang et al. reported that direct contact is a prerequisite for TNT formation and identified F-actin-based TNTs bridging MSCs and recipient cells. Several authors reported that mitochondrial dysfunction is associated also with several neurological diseases such as stroke, spinal cord injury and Alzheimer diseases (AD) [171, 172]. Moreover, there are evidence that MT may be a new approach to restoring mitochondrial functions and that the MT can be used to correct a range of problems caused by mitochondrial dysfunction [173, 174].

### MT transfer and neuronal diseases

In several retinal and corneal diseases has been shown that mitochondrial dysfunction is a critical phenomenon [92, 175]. For example, it has observed that the increase of mitochondrial fission and mitochondrial DNA damage in retinal vasculature precede apoptosis of retinal endothelial cells in diabetic retinopathy [176, 177]. Therefore, several authors thought that targeting mitochondrial dysfunction may be an approach to prevent the development and progression of both retinal and corneal degeneration. Jiang et al. showed that intercellular mitochondrial transport is a vital mechanism for regeneration of corneal epithelial cells and retinal ganglion cells [51, 178]. Moreover, Jiang et al. describe intercellular mitochondrial transportation from MSCs to corneal endothelial cells, photoreceptors, and retinal pigment cells. In this paper they showed that the cell that receive the mitochondrion increased respiratory abilities and elevated expression of mitochondrial structure and function related gene [170]. Mitochondrial dysfunction has observed in Leber’s hereditary optic neuropathy (LHON). Recent evidence showed that LHON derives from a genetic mutation in mitochondria, which evolves to optic atrophy, which gives rise to visual acuity and blindness. Thus, the replacement of mitochondria may be a possible solution, which can be achieved by MT utilizing mesenchymal stem cells or their conditioned media derivate [179]. Several authors investigated a possible role of MT in AD.

AD is a chronic neurodegenerative disease and manifests symptoms such as: short-term memory loss, visual-spatial perception disorders and impaired language and executive functions [180]. Several papers showed that MSCs could inhibit amyloid β-peptide (Aβ) generation and promote its effective clearance, alter amyloid precursor protein (APP) processing, decrease tau phosphorylation, and increase proteasomal activity resulting in reduced accumulation of ubiquitin-conjugated proteins [181–183]. In AD has been observed the presence of Aβ in the mitochondria. This presence of Aβ generates hyperphosphorylated tau proteins by interacting with mitochondrial Drp1 protein, which, in turn, disrupts microtubule function and induces neural toxicity [184, 185]. Moreover, mitochondrial Aβ can also interact with Aβ-binding alcohol dehydrogenase (ABAD), leading to mitochondrial dysfunction and the production of ROS [186]. The main consequence of mitochondrial dysfunction, ROS accumulation, and increased oxidative stress are main factors involved in AD pathogenesis. Several studies showed that MSCs may promote microglia and autophagy-mediated clearance of protein aggregates as Aβ [187–189]. MSCs can protect neurons from cell death by secretion of some neuroprotective factors or by MT [52, 190, 191]. Zhan et al. showed that UC-MSC-CM significantly decreased tau phosphorylated at the Thr181 level, which increased in AD-alleviated intracellular and mitochondrial oxidative stress of okadaic acid (OA)-treated SH-SY5Y cells. In addition, UC-MSC-CM suppressed apoptosis and improved mitochondrial function in OA-treated SH-SY5Y cells. In this paper, Zhan et al. showed that UC-MSC-CM exerted protective effects relying on or partly extracellular vesicle (EV) MT from UC-MSCs to OA-treated SH-SY5Y cells [191]. Moreover, it observed that UC-MSC-CM decreased the level of p181-tau in the AD cell model, improved cell viability, and suppressed apoptosis in OA-treated Sh-SY5Y cells. In addition, they showed that UC-MSC-CM improved mitochondrial functions in OA-treated SH-SY5Y cells [192].

Spinal cord injury (SCI) is a destructive neurological disease that causes major motor, sensory and autonomic dysfunction that in some cases can lead blood vessels rupture and vasoconstriction reflexive led to an oxygen reduction and consequent mitochondrial damage. In addition, it is possible to observe a series of damages such as mitochondrial permeability, calcium overload, excitatory toxicity, oxidative stress, and increased ROS production [193]. These phenomenon results in impaired capability to maintain mitochondrial homeostasis with less energy available [194]. In this context, several authors are evolving new strategies to improve secondary injuries such as repairing or replacing damaged mitochondria, the use of antioxidants, and restoring mitochondrial permeability [195]. Li et al. showed that either MSCs or MSC-derived mitochondria injected into the injured spinal cord of a rat contusion SCI model significantly improved locomotor functions 6 weeks after injury [50]. Other authors reported that MSCs are able to improve the secondary injury caused by inflammation, myelin insulation, and assist the angiogenesis process [9, 196–199]. Sykova et al. showed the safety of the use of intravenous and intraarterial delivery of MSCs in SCI patients [200], while Deng et al. used MSCs coupled with collagen in SCI patients and compared with the control group (only collagen) [201]. After 12 months the study showed the treatment group vs control group showed significantly improved American Spinal Injury Association scores and better bowel and urinary functions [44].The ability of cells to interact with other cells via mitochondria has been demonstrated also in other tissues and organs where different modes of MT from MSCs to injured or damaged cells in order to restore or support nonfunctional mitochondria has been discovered [168]. For example, the active transfer from adult stem cells and somatic cells can rescue aerobic respiration in mammalian cells with non-functional mitochondria [36, 168].

### MT transfer and ischemic vascular diseases

Another pathology that may benefit from studies on stem cell-mediated MT is stroke and ischemia–reperfusion injury. A stroke occurs when something blocks the blood supply to part of the brain or when a blood vessel in the brain bursts. Therefore, we can identify two types of strokes: ischemic or hemorrhagic. The blockage of one or more arteries is the main characteristic of acute ischemic stroke, with blood flow reduction and cellular dysfunction, damage, and/or death. The revascularization process is necessary for stroke treatment, but the oxygen and nutrient trasport to the damaged tissues may lead to the activation of the innate and adaptive immune responses that may cause secondary damage to the remaining cells [202, 203]. The hallmark of ischemia/reperfusion process is a mitochondrial dysfunction, ATP production decrease, ROS increase and cellular death [204]. During this process, cell switchs to anaerobic metabolism with a consequent indirect increase of Ca^2+^ [205]. This Ca^2+^ overload and oxidative stress lead to the opening of mitochondrial permeability transition pore in the inner mitochondrial membrane and an increase in ROS production [206, 207]. Therefore, MT from other cells could represent a useful tool in management of pathological damage caused by mitochondrial dysfunction in ischemia–reperfusion injury. It has been shown that several brain cells as neurons, astrocytes, endothelial cells, and MSCs are able to transfer mitochondria [171]. In preclinical studies, Liu et al. showed that human bone marrow MSCs could save endothelial cells during hypoxia and nutrients deprivation-induced stress. MSCs could abolish apoptosis in the endothelial cells induced by dysfunctional mitochondria during hypoxia by shifting the functional mitochondria from MSCs through TNTs-like cell protrusions [204]. Babenko et al. showed that BM-MSCs are able to save astrocytes and PC12 cells during hypoxia and glucose deprivation induced by oxidative stress and mitochondrial damage. Moreover, the same author reported that in the same in vivo study BM-MSCs improve the neurological impairments of cerebral ischemia rats and that this phenomenon is mediated by MT from BM-MSCs via TNTs. Moreover, it has been reported that BM-MSCs with overexpression of Miro1 improve the recovery of ischemic rats [115, 208]. Transplantation of healthy mitochondria has been studied as a solution in rescuing injured cells and tissue for treatment of others different pathologies connected to ischemia vascular diseases. The first studies have been conducted in animal models of ischemia–reperfusion [209]. For example, Mc Cully et al. have been demonstrated that administering mitochondria isolated from the left ventricle of the rabbit to the site of partial ischemia–reperfusion allowed a significant reduction of myocardial infarction and apoptosis markers with a consequent recovery of myocardial infarction [210]. Then, Masuzawa et al. demonstrated in their work the internalization of the transplanted mitochondria and enhanced myocardial energetics despite the lack of demonstration of internalization (by tunneling nanotubes) mechanism [211]. Similar results have been obtained in rat models of ischemia reperfusion. Kaza et al., administering mitochondria in in vivo rat models prior to reperfusion, obtained a decrease of Infarct Size and Area at Risk (IS/AAR) index and altogether enhanced myocardia and cell viability [212]. Similarly, Guariento and coworkers and Blitzer et al. investigated on the therapeutic use single or multiple intracoronary doses of isolated mitochondria before ischemia–reperfusion episodes [213, 214]. The first clinical application of mitochondrial transplantation was carried out to treat myocardial ischemia–reperfusion injury in pediatric patients of Boston Children’s Hospital (United States) [215]. The role of MT has been demonstrated also in SENECA trials where induced patients derived cardiomyocytes (iCM) co-cultured with MSCs, thanks to MSCs EV release, improved their viability and physiology, reducing ROS production and preserving mitochondrial biogenesis. Moreover, some authors suggested a mechanism by which EV from MSCs resulted enriched in mitochondria that probably were transferred to iCM [216]). Also Liu and colleagued have been demonstrated a mechanism by which MSCs can be used as a novel treatment of ischemic vascular disease; they demonstrated a transfer of mitochondria via a tunnelling nanotube-like structure from stem cells to injured human umbilical vein endothelial cells [204].

### MT transfer and respiratory and pulmonary diseases

The effect of dysfunctional mitochondria has been reported also in respiratory and pulmonary diseases. Among these, Chronic Obstructive Pulmonary Disease (COPD) induced by cigarette smoke (CS) is an example of lung diseases characterized by inflammation and damage of cells. In particular, cigarette smoke induces mitochondrial disfunctions in lung epithelial cells. MSCs and MSC-derived exosomes have been proposed as therapeutic intervention of COPD induced by cigarette smoke as reported by Maremanda et al. which demonstrated as BEAS2B-mMSC co-cultures showed protective response against the CSE-altered mitochondrial respiration parameters, confirming the beneficial effect of MSC towards human bronchial lung epithelial cells [217]. Morrison et al. studying the mechanisms of MSCs effects in ARDS demonstrated that MSCs are able to induce in this environment an anti-inflammatory and highly phagocytic macrophage phenotype through EV-mediated MT. In particular, alveolar macrophage treated with MSC-derived EVs ameliorate lung injury in vivo. Transfer of functional mitochondria via EVs determined an increase in Oxidative phosphorylation which led to enhanced phagocytosis and a decrease of TNF-a and IL-8 levels of secretion by macrophages in vitro and in vivo [140]. Similarly, Jackson et al. reported that human bone marrow derived MSCs transfer their mitochondria to macrophages both in vivo and in vitro via TNT and micro vesicle secretion. This leads to enhanced macrophage phagocytosis and improved bioenergetics. So mitochondrial donation represented a novel mechanism to explain the antimicrobial effect of MSCs in a condition determined by bacterial infection providing additional evidence about their therapeutic use in acute, inflammatory lung diseases [64]. Moreover, the effect of mitochondrial transplantation or donation has been reported in other cell pathologies including renal and liver diseases. For example, Kubat et al. observed good therapeutic effects of mitochondrial transplantation in a nephrotoxicity model [218]. Otherwise, Lu et al. demonstrated a nanotherapeutic effect of UC-MSC-EVs on inhibiting local NETs formation by transferring functional mitochondria to intrahepatic neutrophils and repairing their mitochondrial function [219]. In addition, Bi et al. reported a functional therapeutic effect of mitochondrial transfer to combat Non-alcoholic fatty liver disease [220]. By the way, MT has been observed in lung diseases also associated with bioenergetic impairment and dysfunctional mitochondria such as allergic airway inflammation, ARDS or asthma but what signalling events can trigger this mechanism remain elusive.

## Conclusions

Mitochondrial transfer and the mechanisms by which mitochondria enter recipient cells are concepts that have gained much attention in recent years. In particular, intercellular communication and mitochondrial transfer in MSCs promise interesting therapeutic results in various pathological conditions. In general, the active transfer of mitochondria from mesenchymal cells to somatic cells could, for instance, restore aerobic respiration in cells with non-functional mitochondria. So, the possible transplantation of healthy mitochondria has been studied as a solution in rescuing injured cells and tissue for treatment of several pathologies such as pulmonary, cardiac, renal, corneal epithelium, and brain cortical. Therefore, understanding the molecular and cellular mechanisms of mitochondrial transfer/transplantation and demonstrating its efficacy could be an important milestone that lays the foundation for future clinical trials.

## Data Availability

Not applicable.

## Abbreviations

Arp2/3: Actin related protein 2/3 complex
AML: Acute myeloid leukemia
ARDS: Acute respiratory distress syndrome
AD: Adipose
AD: Alzheimer diseases
BM: Bone marrow
CSF: Cerebrospinal fluid
COPD: Chronic Obstructive Pulmonary Disease
CX43: Connexin 43
Cyt-C: Cytochrome C
DAMPs: Damage-associated molecular patterns
DP: Dental pulp
ER: Endoplasmic reticulum
EVs: Extracellular vesicles
GJs: Gap junctions
HSCs: Haematopoietic stem cells
iPSC: Human induced pluripotent stem cell
HTMCs: Human trabecular meshwork cells
IRSp53: I-Bin/Amphiphysin/Rvs (BAR)-domain protein
iTNTs: Individual TNTs
iCM: Induced patients derived cardiomyocytes
LHON: Leber’s hereditary optic neuropathy
MSCs: Mesenchymal stem cells
MVs: Microvesicles
MICOS: Mitochondrial contact-site and cristae organizing system
mtDNA: Mitochondrial DNA
MQC: Mitochondrial quality control
MT: Mitochondrial transfer
MDRCs: Myeloid-derived regulatory cells
NOX2: NADPH oxidase-2
NCDs: Non‐communicable diseases
OMM: Outer mitochondrial membrane
PI(4;5)P_2_: Phosphatidylinositol (4;5)-bisphosphate
ROS: Reactive oxygen species
RAGE: Receptor for Advanced Glycation End Product
SCI: Spinal cord injury
TNFαip2: TNF-α-induced protein 2
TFAM: Transcription factor A mitochondria
TEC: Tubular epithelial cells
TNF: Tumor necrosis factor
TNTs: Tunnelling nanotubes
UC: Umbilical cord
WAVE2: WASP family verprolin-homologous 2
WJ: Wharton’s jelly
